# Trends and influence factors in the prevalence, intervention, and control of metabolic syndrome among US adults, 1999–2018

**DOI:** 10.1186/s12877-022-03672-6

**Published:** 2022-12-19

**Authors:** Chaojun Yang, Xiaocan Jia, Yuping Wang, Jingwen Fan, Chenyu Zhao, Yongli Yang, Xuezhong Shi

**Affiliations:** Department of Epidemiology and Biostatistics, College of Public Health, Zhengzhou University, Zhengzhou, 450001 Henan China

**Keywords:** Metabolic syndrome, Hyperglycemia, Hypertension, Dyslipidemia, Obesity

## Abstract

**Aim:**

We aimed to describe the trends in the prevalence, intervention, and control of metabolic syndrome (MetS) among US adults through 1999–2018. Additionally, the influence factors of MetS and its control were further explored.

**Methods:**

We included participants older than 20 using the National Health and Nutrition Examination Survey (NHANES) from 1999 to 2018 (*n* = 22,114). The rate of prevalence, intervention, and control of MetS were caculated by survey weights. Joinpoint regression and survey-weighted generalized linear models were used to analyze trends and influence factors, respectively.

**Results:**

The prevalence of MetS increased from 28.23 to 37.09% during 1999–2018 (*P* for trend < 0.05). The former smoker (OR = 1.20, 95%CI: 1.07, 1.36) and current smoker (OR = 1.27, 95%CI: 1.11, 1.45) increased the prevalence of MetS. While vigorous activity (OR = 0.53, 95%CI: 0.47, 0.61) decreased it. Among MetS components, the prevalence of elevated blood-glucose (from 21.18 to 34.68%) and obesity (from 44.81 to 59.06%) raised (*P* for trend < 0.05), with an uptrend in the use of antidiabetic (from 9.87 to 28.63%) and a downtrend of vigorous activity (from 23.79 to 16.53%) (*P* for trend < 0.05). Decreased trends were observed in the control of Hb1Ac (< 7%) (from 87.13 to 84.06%) and BMI (<25 kg/m^2^) (from 11.36 to 7.49%). Among MetS underwent antidiabetic, 45–64 years old and male decreased the control of Hb1Ac (< 7%). The control of BMI (<25 kg/m^2^) among individuals with physical activity was reduced mainly in the population of younger (aged 20–44 years old), male, non-Hispanic black, middle income and smoker (former and current).

**Conclusions:**

The prevalence of MetS increased significantly through 1999–2018. Elevated blood glucose and obesity were the main causes of MetS burden. Quitting smoking and increasing physical activity may decrease the prevalence of MetS. In the control of blood-glucose and obesity, we should screen out the focus population to modify treatment and improve lifestyle.

**Supplementary Information:**

The online version contains supplementary material available at 10.1186/s12877-022-03672-6.

## Introduction

Metabolic syndrome (MetS) is a constellation of metabolic abnormalities characterized by hyperglycemia, dyslipidemia, hypertension, and abdominal obesity [1]. Each component of MetS may increase the risk of cardiovascular disease occurrence [2], diabetes developing [3], stroke recurrence [4], and all-cause mortality [5]. In the 2022 statistical update of heart disease and stroke [6], the prevalence of cardiovascular disease (including coronary heart disease, heart failure, stroke, and hypertension) in adults ≥20 years old was 49.2% overall; meanwhile, an average of 19 million deaths was attributed to cardiovascular disease globally in 2020. Thus, it is crucial for the prevention and control of cardiovascular diseases to understand trends in the prevalence of MetS and the status of its intervention and control.

The prevalence of diabetes increased significantly from 9.8% in 1999–2000 to 14.3% in 2017–2018, with a decline in glycemic control (Hb1Ac < 7%) from 57.4 to 50.5% among the adult National Health and Nutrition Examination Survey (NHANES) participants with diabetes [7, 8]. The estimated US prevalence of severe dyslipidemia was 6.6%, while the statin use among them was uniformly low at 37.6 % [9]. In 2019, the global age-standardized prevalence of hypertension in adults aged 30–79 years was 32% in women and 34% in men, among whom the control rate was only 23 and 18%, respectively [10]. From 1999 to 2000 to 2017–2018, the prevalence of obesity increased from 30.5 to 42.4% among adults [11]. Previous studies reported that the prevalence of MetS was 23.7% in 1988–1994 and 23% in 2003–2014 among US adults [12, 13]. Trend analysis showed that the prevalence of MetS decreased from 25.5 to 22.9% during 1999–2010, and subsequently increased from 32.5 to 36.9% through 2011–2016 in the US adult [14, 15]. During 1999–2018, US cardiometabolic health, defined by optimal levels of blood glucose, blood lipids, blood pressure, and adiposity, has been poor and worsening, with only 6.8% of adults having optimal cardiometabolic health [16], which may reflect an unoptimistic status of MetS.

The influence factors of MetS and its control are very important for the improvement of MetS burden. Chitrala et al. [17] discovered that there was race-specific alteration in DNA methylation among middle-aged African Americans and Whites with MetS. Krijnen et al. [18] found that education was inversely associated with MetS development for males; education, income and occupational prestige were inversely associated with MetS development for females. Besides, therapeutic lifestyle changes, such as a Mediterranean diet and exercise, could decrease the risk of MetS [19]. Thus, demographics characteristics, socioeconomic position and lifestyle are main factors for the prevalence of MetS and its control.

However, there was no estimation of the trend in the prevalence, intervention, and control of MetS over 20 years period in US, especially no update after 2016. Concurrently, there was few studies to explore the influence factors of MetS control after the intervention. The objectives of our study were to update the national trend in the prevalence of MetS and assess the tendency of medication use and metabolic risk factors control in MetS among US adults. Furthermore, we explore the influence factors of MetS and its control after the intervention.

## Material and methods

### Data collection

The NHANES is a complex, multistage sampling design and nationally representative survey to monitor the health of the US population conducted by the Centers for Disease Control and Prevention [20]. Participants were recruited from the US non-institutionalized, civilian population, who underwent 4 stages of selection: counties, segments, households, and individuals. Data were collected through in-home interviews and study visits to a mobile examination center (MEC), releasing in every 2-year. The study protocols of NHANES were approved by the NCHS Research Ethics Review Board and obtained participants’ written informed consent [21].

Our study included the participants in the NHANES from 1999 to 2018 who were more than 20 years old and nonpregnant. We selected individuals who fasted for 8 h or more and had complete information on glucose, HDL, triglycerides, blood pressure, and waist circumference. We excluded persons with no information on sampling design parameters, including primary sampling units (PSUs), stratum, and fasting subsample weight.

### Definition of metabolic syndrome

MetS was defined based on the National Cholesterol Education Program’s Adult Treatment Panel III (ATP III) as having 3 or more of the following [22]: 1) fasting plasma glucose level at least 5.6 mmol/L (or 100 mg/dL) or drug treatment for elevated blood glucose; 2) high-density lipoprotein (HDL) cholesterol less than 40 mg/dL (or 1.0 mmol/L) in men or less than 50 mg/dL (or 1.3 mmol/L) in women or drug treatment for low HDL; 3) triglyceride level greater than 150 mg/dL (or 1.7 mmol/L) or drug treatment for elevated triglyceride; 4) waist circumference greater than 102 cm in men or 88 cm in women; 5) systolic blood pressure at least 130 mmHg, diastolic blood pressure at least 85 mmHg or taking hypertension medications.

### Medication use

During the household interview, survey participants were asked if they had taken any prescription medications in the past 30 days. For those who answered “yes”, the medication containers of all the products used should be shown to the interviewer. If containers were unavailable, participants could report the name of the medication. When the interviewer entered the medication name into the computer, the name was automatically identified as an exact match or similar text matches [23]. The usage of antidiabetic (yes or no), antihyperlipidemic (yes or no), and antihypertensive (yes or no) were obtained by drug-classification codes.

### Metabolic risk factors control

The metabolic risk factors and control standards were based on the diagnosis and the management of MetS in ATP III [22], including glycated hemoglobin (HbA1c), fasting blood-glucose, triglyceride, HDL, blood pressure, and body mass index (BMI). The hematological indicators were measured following the NHANES protocol. Blood pressure was measured on the same arm three consecutive times with a mercury sphygmomanometer, calculated for average. BMI was calculated as weight in kilograms divided by height in meters squared. We defined glycemic control as HbA1c <8.0% and <7.0%, and fasting blood-glucose <5.6 mmol/L (or 100 mg/dL). Lipid control was regarded as HDL ≥ 40 mg/dL (or 1.0 mmol/L) in men and ≥ 50 mg/dL (or 1.3 mmol/L) in women, and triglyceride <150 mg/dL (or 1.7 mmol/L). Blood pressure control was ruled by <140/90 mmHg and a more stringent target of <130/80 mmHg. Obesity status was stated by BMI: thin (< 18.5 kg/m^2^), normal weight (18.5–24.9 kg/m^2^), overweight (25.0–29.9 kg/m^2^), and obesity (≥30.0 kg/m^2^) [24].

### Social demography and lifestyle characteristics

Participants reported sociodemographic factors, including age, sex (male and female), race (non-Hispanic white, non-Hispanic black, Mexican American, Non-Hispanic Asian and other race), marital status (yes and no), education level (less than high school, high school graduate, some college, and college graduate or above), insurance status (uninsured and any insurance), income-to-poverty ratio (PIR) (< 1, 1–2, 2–3.5 and ≥ 3.5) [25]. Lifestyle information was obtained from questionnaires, including alcohol, smoking status, and activity. The activity was divided into three levels: vigorous, moderate and no. The alcohol use was divided into two categories: yes (more than 12 drinks) and no. The smoking status was grouped: never smoked (smoked less than 100 cigarettes in life); former smoker (smoked at least 100 cigarettes in life and not at all now); current smoker (smoked at least 100 cigarettes in life and smoked some days or every day now).

### Statistical analysis

NHANES data was extracted and preprocessed by “nhanesR package”. NHANES provided weights to ensure the representative and unbiased estimation of the total civilian noninstitutionalized US population. Fasting subsample weight was selected in this study. The prevalence and proportion were weighted by 2-year weights, as well as the characteristics distribution. While, we combined 20-year weights to calculate the total prevalence or proportion, and analyze the influence factors.

The estimation of weighted prevalence and proportion were both conducted by “survey package” that was specially handled complex sample design surveys, summarized as mean and 95% confidence interval (CI). Joinpoint regressions were used to calculate average annual percent change (AAPC) and its 95% CI, determining trends in log-transformed prevalence, intervention, and control of MetS, allowing 1 joinpoint. The Monte Carlo method was used to select the best-fitting model and identify the point of change in trends (joinpoint) [26]. When the AAPC and its 95% CI were both greater than 0, the rate indicated an uptrend; on the contrary, when the AAPC and its 95% CI were both less than 0, the rate indicated a downtrend; any AAPC with a 95% CI overlapping with zero was considered stable trend.

Rao-Scott *χ*^2^ test was used for assessing the discrepancy of distribution over time in sociodemographic and lifestyle factors. Survey-weighted generalized linear models were performed to evaluate odds ratio (OR) and 95% CI by “survey package” (family = quasibinomial), estimating the factors associated with the prevalence of MetS, and the factors for the control of MetS after the intervention.

Statistical analyses were performed with R version 4.1.1 software and Joinpoint regressions Program 4.9.0.0. Statistical significance was < 0.05.

## Result

### Trend in the prevalence of MetS

**Fig. 1:**
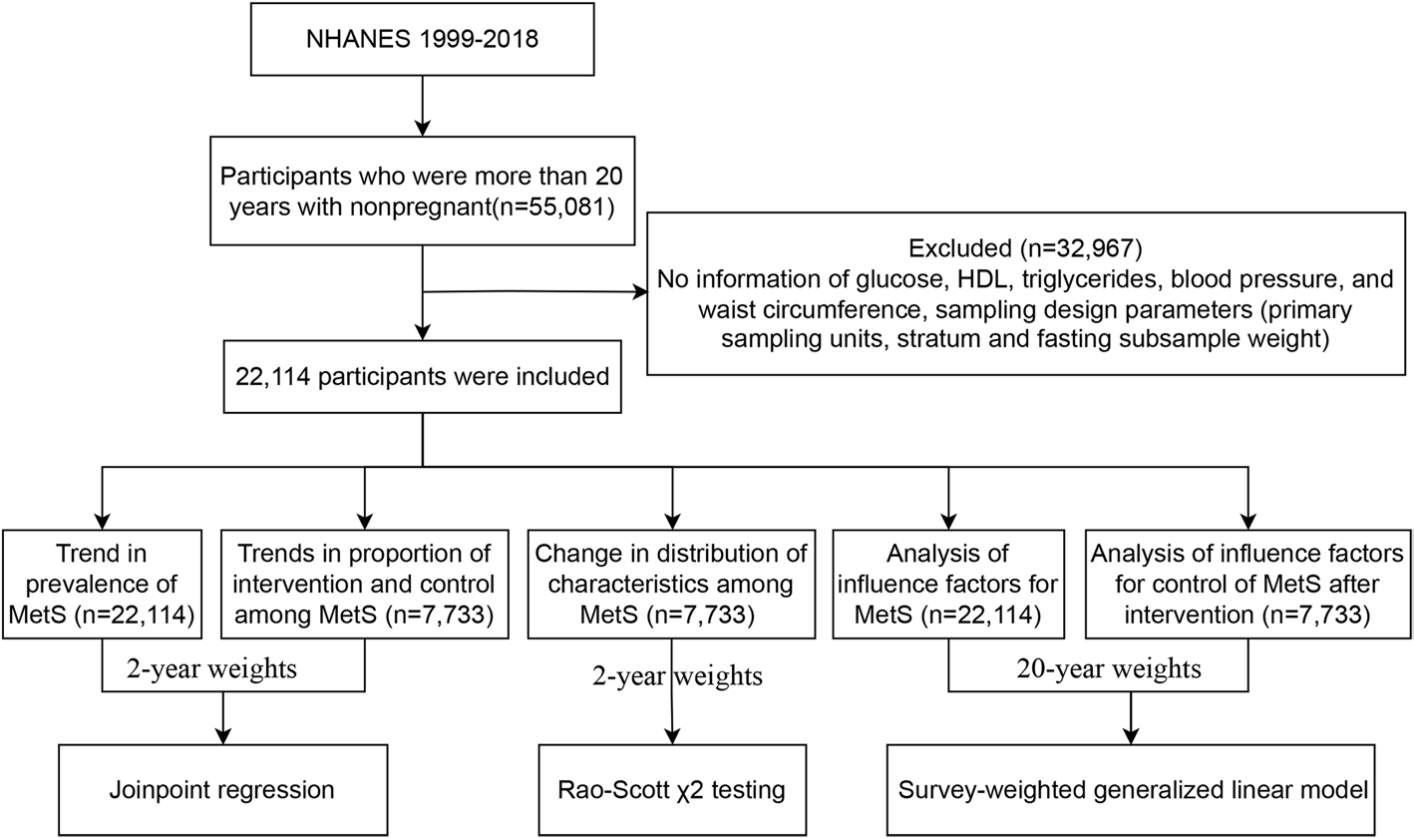
Flow chart

**Fig. 2:**
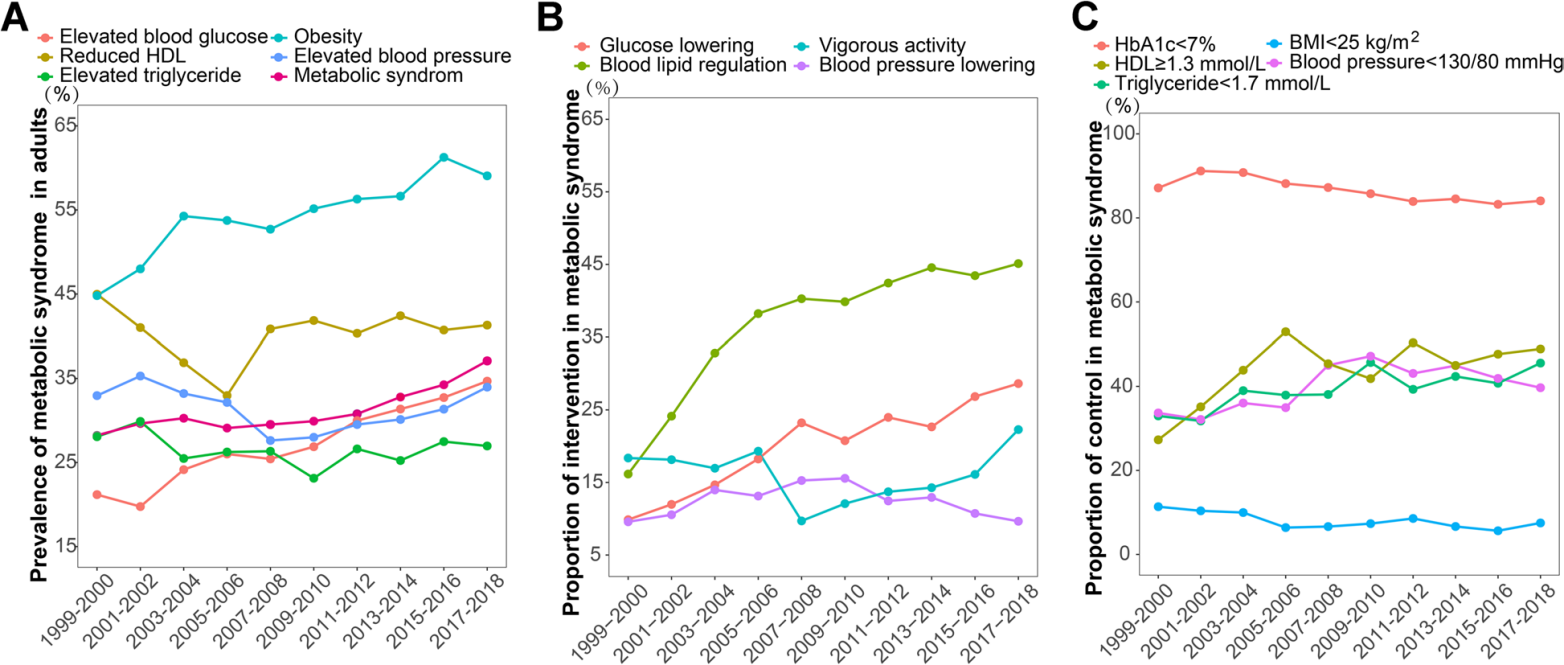
Trend in prevalence of intervention and control among metabolic syndrome from 1999 to 2018. (A) Prevalence of metabolic syndrome in adults. (B) Proportion of intervention in metabolic syndrome. (C) Proportion of control in metabolic syndrome

For analyzing the trend in the prevalence of MetS, 22,114 participants were included, representing an estimated 203,067,836 US adults aged 20 or older (Fig. 1; sTable1). A total of 7733 patients satisfied the diagnosis criteria of MetS, with a prevalence of 31.43%. The prevalence of MetS components, elevated blood glucose, reduced HDL, elevated triglyceride, obesity, and elevated blood pressure were 27.61, 40.32, 26.58, 54.57, and 31.61%, respectively. The estimated prevalence of MetS increased significantly from 28.23 to 37.09% from 1999 to 2018, with a 1.385% (95% CI: 0.778, 1.996%) relative increase per 2-year cycle (Table 1; Fig. 2 A; A significant increase in the estimated prevalence of MetS was observed in the following population over the study period: more than 65 years old (from 50.78 to 63.02%; *P* <  0.001 for trend), non-Hispanic white adults (from 28.32 to 38.33%; *P* = 0.003 for trend), Mexican American adults (from 25.36 to 40.64%; *P* = 0.005 for trend), married (from 29.44 to 41.14%; *P* = 0.016 for trend) or not (from 27.11 to 32.67%; *P* = 0.003 for trend), insured adults (from 29.29 to 38.56%; *P* = 0.033 for trend), alcohol (from 25.44 to 36.91%; *P* <  0.001 for trend) and no activity (from 34.95 to 44.53%; *P* = 0.035 for trend). The inflection point, the annual rate of change before or after inflection, and AAPC in the prevalence of MetS were shown in Additional file 3 sTable 2.

**Table 1:** Trends in prevalence of metabolic syndrome among US adults from 1999 to 2018

Characteristics		1999–2000 (*n* = 1948)	2001–2002 (*n* = 2187)	2003–2004 (*n* = 1945)	2005–2006 (*n* = 2005)	2007–2008 (*n* = 2406)	2009–2010 (*n* = 2564)	2011–2012 (*n* = 2245)	2013–2014 (*n* = 2362)	2015–2016 (*n* = 2263)	2017–2018 (*n* = 2189)	AAPC	*P* for trend
Total		28.23(25.85,30.60)	29.64(27.35, 31.93)	30.27(27.45, 33.08)	29.10(26.19, 32.02)	29.52(27.28, 31.77)	29.92(27.00, 32.85)	30.78(27.66, 33.89)	32.79(30.11, 35.48)	34.25(31.15, 37.36)	37.09(33.66, 40.51)	1.385(0.778, 1.996)	< 0.001
Age (years)													
	20–44	17.94(14.60, 21.27)	16.49(14.20, 18.78)	17.90(15.26, 20.55)	16.51(13.53, 19.48)	16.56(13.04, 20.07)	16.27(12.13, 20.40)	15.82(12.20, 19.44)	18.34(15.01, 21.66)	19.51(15.21, 23.82)	20.49(16.53, 24.45)	1.015(−0.195, 2.239)	0.100
	45–64	36.75(31.46, 42.04)	40.30(35.59, 45.00)	38.55(33.86, 43.25)	34.83(30.04, 39.62)	34.99(31.86, 38.13)	36.39(33.17, 39.62)	37.49(31.03, 43.95)	40.24(35.40, 45.09)	41.10(35.39, 46.80)	42.50(35.98, 49.03)	0.457(−0.721, 1.650)	0.448
	≥65	50.78(43.17, 58.40)	53.12(47.25, 59.00)	52.19(46.40, 57.98)	52.82(46.30, 59.34)	54.07(46.88, 61.27)	52.96(48.77, 57.16)	57.57(49.91, 65.23)	55.62(49.81, 61.44)	54.80(50.14, 59.45)	63.02(58.30, 67.75)	0.968(0.251, 1.690)	0.008
Sex													
	Female	30.14(27.94, 32.35)	29.59(26.73, 32.46)	29.48(24.87, 34.10)	28.10(23.55, 32.66)	30.11(26.97, 33.24)	28.70(25.40, 31.99)	30.48(26.56, 34.41)	32.83(29.13, 36.54)	32.46(28.44, 36.47)	35.58(31.46, 39.70)	0.223(−0.821, 1.279)	0.124
	Male	26.17(21.92, 30.42)	29.70(25.96, 33.43)	31.07(26.76, 35.37)	30.18(26.99, 33.37)	28.91(25.88, 31.94)	31.19(26.23, 36.15)	31.08(27.61, 34.55)	32.75(29.54, 35.97)	36.16(31.91, 40.40)	38.65(33.82, 43.49)	1.269(−0.345, 2.909)	0.676
Race and ethnicity													
	Non-Hispanic white	28.32(24.84, 31.80)	30.14(27.94, 32.34)	32.29(28.26, 36.32)	30.70(27.00, 34.40)	31.00(27.41, 34.60)	30.64(27.26, 34.03)	31.62(27.58, 35.65)	35.24(31.71, 38.76)	36.41(32.26, 40.55)	38.33(33.42, 43.24)	1.438(0.493, 2.392)	0.003
	Non-Hispanic black	21.42(17.67, 25.17)	27.05(22.16, 31.94)	21.73(17.70, 25.75)	26.43(22.03, 30.84)	26.49(19.36, 33.63)	29.96(23.83, 36.10)	30.33(26.85, 33.81)	29.70(24.20, 35.20)	28.24(25.17, 31.31)	29.59(24.53, 34.65)	1.333(−1.272, 4.007)	0.319
	Mexican American	25.36(18.04, 32.68)	27.90(21.85, 33.94)	28.21(20.67, 35.75)	24.72(18.16, 31.28)	28.23(24.18, 32.28)	32.36(27.04, 37.69)	32.92(25.80, 40.03)	29.32(23.90, 34.73)	36.91(32.04, 41.77)	40.64(35.33, 45.96)	2.580(0.771, 4.422)	0.005
	Other Hispanic	35.07(27.78, 42.36)	31.57(10.54, 52.60)	17.57(2.47, 32.68)	19.18(9.75, 28.61)	26.25(21.43, 31.06)	28.00(24.09, 31.91)	29.64(23.47, 35.81)	26.24(19.37, 33.11)	28.79(22.37, 35.21)	34.42(25.43, 43.41)	−0.494(−6.343, 5.721)	0.873
	Other race	33.24(16.27, 50.21)	28.45(16.59, 40.30)	29.83(20.67, 38.99)	26.22(11.06, 41.38)	21.85(13.22, 30.47)	20.76(11.66, 29.86)	22.75(14.30, 31.19)	25.18(19.59, 30.78)	28.41(22.08, 34.74)	35.94(28.00, 43.87)	−0.068(−1.850, 1.745)	0.941
Marital status													
	No	27.11(21.98, 32.24)	26.86(21.96, 31.75)	27.17(22.79, 31.54)	22.98(19.55, 26.41)	27.14(23.64, 30.65)	28.40(23.76, 33.04)	27.93(23.97, 31.89)	30.15(25.36, 34.94)	31.54(27.81, 35.27)	32.67(29.63, 35.70)	0.938(0.319, 1.561)	0.003
	Yes	29.44(24.84, 34.04)	31.44(28.79, 34.09)	32.60(28.60, 36.60)	33.50(29.22, 37.78)	31.28(28.11, 34.45)	30.92(27.55, 34.30)	33.16(29.35, 36.97)	34.76(30.87, 38.65)	36.40(32.04, 40.76)	41.14(35.57, 46.71)	1.428(0.263, 2.606)	0.016
Education level													
	Less than high school	31.83(25.07, 38.58)	39.47(33.23, 45.71)	31.83(28.05, 35.61)	35.09(29.51, 40.67)	38.52(34.02, 43.02)	40.27(35.81, 44.74)	36.81(32.61, 41.01)	38.41(32.90, 43.92)	38.87(33.13, 44.61)	46.10(39.42, 52.79)	1.521(−1.781, 4.933)	0.371
	High school graduate	32.35(25.51, 39.20)	32.34(27.50, 37.18)	36.23(31.28, 41.18)	35.85(31.72, 39.98)	33.89(27.50, 40.29)	35.76(31.38, 40.14)	36.88(32.69, 41.08)	35.76(28.94, 42.58)	36.54(29.51, 43.56)	43.21(35.22, 51.19)	1.229(−0.460, 2.947)	0.155
	Some college	31.09(23.31, 38.87)	24.95(21.61, 28.29)	28.98(25.53, 32.43)	29.10(24.83, 33.37)	27.33(23.13, 31.53)	31.16(25.62, 36.69)	30.31(25.04, 35.59)	36.85(32.79, 40.91)	35.48(29.68, 41.29)	36.18(31.79, 40.56)	1.914(−0.577, 4.468)	0.133
	College graduate or above	16.18(10.45, 21.92)	25.54(21.05, 30.02)	24.43(18.47, 30.38)	18.21(12.91, 23.51)	21.85(17.61, 26.10)	17.41(13.44, 21.38)	24.07(18.18, 29.97)	23.76(19.39, 28.13)	29.38(23.14, 35.62)	28.83(24.31, 33.36)	1.234(−2.484, 5.093)	0.521
Insurance status													
	Uninsured	24.50(17.25, 31.75)	15.79(11.41, 20.17)	24.20(18.43, 29.97)	20.57(14.72, 26.41)	22.75(18.41, 27.09)	24.21(19.38, 29.03)	24.91(21.08, 28.74)	21.79(17.97, 25.61)	26.92(22.57, 31.27)	27.90(20.64, 35.17)	1.788(−2.588, 6.360)	0.429
	Any Insurance	29.29(26.47, 32.11)	32.49(30.10, 34.88)	31.63(28.59, 34.66)	31.11(27.87, 34.35)	31.01(28.06, 33.95)	31.52(28.07, 34.98)	32.25(28.77, 35.74)	35.22(31.73, 38.71)	35.36(31.73, 38.98)	38.56(35.25, 41.87)	1.137(0.094, 2.191)	0.033
Income-to-poverty ratio													
	< 1	28.30(23.14, 33.47)	32.63(23.86, 41.41)	24.29(19.43, 29.15)	32.63(22.23, 43.03)	32.08(27.58, 36.58)	28.48(23.80, 33.15)	28.35(23.77, 32.94)	34.74(29.41, 40.07)	33.01(26.88, 39.14)	36.23(30.93, 41.53)	1.401(−1.158, 4.026)	0.286
	1–2	31.77(25.83, 37.71)	29.53(23.45, 35.60)	33.84(30.00, 37.69)	33.17(26.91, 39.43)	34.95(30.22, 39.68)	34.15(28.41, 39.90)	35.95(30.57, 41.34)	36.10(30.65, 41.54)	34.08(30.28, 37.88)	41.01(35.73, 46.28)	1.064(0, 2.140)	0.050
	2–3.5	34.25(26.73, 41.77)	29.11(22.82, 35.40)	31.86(26.66, 37.06)	29.14(22.73, 35.56)	28.30(23.81, 32.79)	31.92(26.13, 37.72)	32.60(26.38, 38.82)	32.67(26.75, 38.59)	36.03(29.90, 42.16)	40.84(35.60, 46.08)	1.035(− 0.587, 2.683)	0.212
	≥3.5	23.49(18.72, 28.25)	28.61(25.97, 31.25)	28.62(22.66, 34.58)	26.09(22.79, 29.39)	27.49(24.55, 30.43)	26.48(21.82, 31.15)	27.48(21.50, 33.45)	31.04(26.79, 35.28)	33.76(27.20, 40.32)	33.03(27.45, 38.60)	1.22(−0.807, 3.288)	0.240
Alcohol													
	No	36.41(32.01, 40.82)	38.58(30.91, 46.25)	38.64(34.94, 42.35)	37.73(31.64, 43.82)	35.01(30.96, 39.06)	40.33(35.62, 45.04)	38.64(32.89, 44.39)	40.66(35.10, 46.21)	39.30(35.82, 42.78)	42.37(32.56, 52.18)	0.473(−0.314, 1.267)	0.240
	Yes	25.44(22.78, 28.10)	26.51(23.65, 29.37)	27.75(24.55, 30.96)	26.01(22.84, 29.18)	27.60(24.86, 30.33)	27.80(24.43, 31.16)	30.31(26.61, 34.02)	31.17(28.71, 33.63)	32.97(29.42, 36.51)	36.91(33.37, 40.44)	1.859(1.045, 2.678)	< 0.001
Smoking status													
	Never smoker	26.74(22.34, 31.14)	27.26(23.29, 31.23)	28.63(24.55, 32.71)	26.40(22.45, 30.35)	25.94(22.94, 28.94)	27.62(23.81, 31.43)	27.19(24.38, 30.01)	29.61(25.26, 33.96)	29.80(26.09, 33.50)	32.93(27.60, 38.26)	0.996(−0.222, 2.229)	0.109
	Former smoker	34.33(29.17, 39.50)	36.42(30.88, 41.97)	39.74(34.44, 45.04)	37.45(32.31, 42.59)	40.38(36.28, 44.49)	37.00(32.43, 41.57)	37.80(30.78, 44.83)	41.11(37.17, 45.06)	46.37(40.66, 52.08)	44.35(38.55, 50.15)	1.318(−0.245, 2.905)	0.099
	Current smoker	24.63(20.61, 28.66)	27.45(22.87, 32.03)	24.23(20.16, 28.31)	26.05(21.77, 30.32)	25.64(21.44, 29.84)	27.20(20.60, 33.80)	32.72(27.77, 37.66)	31.64(27.78, 35.51)	29.79(24.53, 35.06)	39.35(32.81, 45.88)	2.083(−0.424, 4.652)	0.104
Activity													
	No	34.95(30.37, 39.53)	38.41(34.72, 42.11)	33.03(29.41, 36.65)	39.00(32.94, 45.06)	37.75(35.50, 39.99)	38.69(35.73, 41.65)	38.72(34.14, 43.30)	41.64(38.05, 45.23)	42.29(37.85, 46.73)	44.53(39.10, 49.97)	0.971(0.066, 1.884)	0.035
	Moderate	32.11(26.03, 38.19)	33.84(29.48, 38.20)	39.54(35.72, 43.36)	31.31(26.15, 36.47)	34.09(29.06, 39.12)	31.06(27.28, 34.83)	33.47(27.61, 39.33)	33.74(28.37, 39.11)	37.66(33.61, 41.71)	39.91(33.93, 45.89)	0.515(−2.584, 3.713)	0.956
	Vigorous	18.37(14.49, 22.26)	18.14(14.71, 21.57)	16.96(12.62, 21.29)	19.31(15.67, 22.95)	9.70(6.73, 12.66)	12.08(7.63, 16.52)	13.72(10.10, 17.35)	14.28(11.40, 17.17)	16.11(12.89, 19.33)	22.29(15.99, 28.59)	0.158(−5.289, 5.919)	0.748
BMI (kg/m^2^)^a^													
	Normal weight (18.5–24.9)	8.55(4.98, 12.12)	9.24(6.61, 11.86)	9.45(7.02, 11.88)	5.90(4.41, 7.40)	6.21(4.39, 8.03)	7.44(5.42, 9.46)	8.93(6.49, 11.36)	7.65(4.26, 11.05)	7.74(5.50, 9.98)	10.22(7.29, 13.14)	−0.385(−4.565, 3.979)	0.860
	Overweight (25.0–29.9)	26.27(23.68, 28.86)	31.02(28.16, 33.88)	29.33(24.92, 33.75)	25.14(19.03, 31.25)	27.58(23.79, 31.38)	24.16(21.59, 26.72)	24.33(20.23, 28.42)	31.09(27.65, 34.52)	29.92(25.47, 34.38)	33.46(28.29, 38.64)	0.798(−1.936, 3.609)	0.571
	Obesity (≥30.0)	57.59(53.18, 62.01)	51.28(46.73, 55.83)	53.79(49.27, 58.31)	55.23(51.10, 59.35)	55.78(51.90, 59.65)	54.83(48.40, 61.26)	55.95(51.54, 60.35)	54.43(48.98, 59.88)	55.32(50.41, 60.22)	58.25(53.06, 63.43)	0.09(−0.557, 0.740)	0.787

^a^ 352(underweight) participants with BMI less than 18.5 were not showed; AAPC: average annual rates of change

Among the trends in the prevalence of MetS components, only elevated blood glucose (from 21.18 to 34.68%) and obesity (from 44.81 to 59.06%) were increasing significantly, with 3.337% (95%CI: 2.072, 4.618%) and 1.597% (95%CI: 0.678, 2.526%) relative increase per 2-cycle, respectively, from 1999 to 2000 to 2017–2018 (Additional file 4 sTable 3- Additional file 8 sTable 7, Fig. 2 A). The relative rate change per 2-year cycle in the prevalence of MetS components was displayed in Additional file 3 sTable 2. There was an upward trend both in the prevalence of elevated blood glucose and obesity among the population of 20–44 years old, more than 65 years old, female, non-Hispanic white, non-Hispanic black, Mexican American, all marital status, low education level, any insurance, low economic level, smoking now, no activity, and overweight (all *P* for trend < 0.05).

### Analysis of influence factors for MetS

Univariate analysis showed that the estimated prevalence of MetS was significantly higher in older, non-Hispanic white, married, low level of education, insured adults, middle economic level, didn’t drink alcohol, former smoker, no activity, and obesity (Additional file 9 sTable 8). There was a statistical difference in the prevalence of each component of MetS in sociodemographic and lifestyle factors (*P* <  0.05) (Additional file 9 sTable 8). Establishing survey-weighted generalized linear models, the association between the prevalence of MetS and sociodemographic and lifestyle was estimated (Table 2). After multivariate analysis, the risk factors for MetS were 45–64 years old (OR = 2.75; 95%CI: 2.42, 3.12), more than 65 years old (OR = 6.48; 95%CI: 5.68, 7.39), male (OR = 1.20; 95%CI: 1.08, 1.33), any insurance (OR = 1.28; 95%CI: 1.13, 1.45), former smoker (OR = 1.20; 95%CI: 1.07, 1.36), current smoker (OR = 1.27; 95%CI: 1.11, 1.45), 25 ≤ BMI<30 kg/m^2^ (OR = 4.44; 95%CI: 3.88, 5.09) and BMI ≥ 30.0 kg/m^2^ (OR = 16.49; 95%CI: 14.20, 19.14). The strongest association was found in the more than 65 years old group. While, non-Hispanic black (OR = 0.66; 95%CI: 0.57, 0.75), college graduate or above (OR = 0.70; 95%CI: 0.59, 0.82), PIR ≥ 3.5 (OR = 0.82; 95%CI: 0.69, 0.98), alcohol (OR = 0.77; 95%CI: 0.69, 0.86), and vigorous activity (OR = 0.53; 95%CI: 0.47, 0.61) decreased the prevalence of MetS. The factors associated with the prevalence of MetS components was also shown in Table 2.

**Table 2:** Analysis of influence factors for the prevalence of metabolic syndrome and its components

Factors		MetS	Elevated blood glucose	Reduced HDL	Elevated triglyceride	Obesity	Elevated blood pressure
Age (years)							
	20–44	1(reference)	1(reference)	1(reference)	1(reference)	1(reference)	1(reference)
	45–64	2.75(2.42, 3.12)	3.43(3.05, 3.85)	1.39(1.26, 1.53)	1.34(1.20, 1.50)	2.28(1.94, 2.67)	3.13(2.81, 3.49)
	≥65	6.48(5.68, 7.39)	7.39(6.47, 8.43)	2.52(2.27, 2.81)	1.37(1.23, 1.54)	3.76(3.12, 4.54)	7.22(6.39, 8.17)
Sex							
	Female	1(reference)	1(reference)	1(reference)	1(reference)	1(reference)	1(reference)
	Male	1.20(1.08, 1.33)	1.86(1.70, 2.03)	0.98(0.89, 1.08)	1.41(1.27, 1.56)	0.10(0.08, 0.12)	1.47(1.34, 1.61)
Race and ethnicity							
	Non-Hispanic white	1(reference)	1(reference)	1(reference)	1(reference)	1(reference)	1(reference)
	Non-Hispanic black	0.66(0.57, 0.75)	1.14(1.01, 1.29)	0.62(0.55, 0.70)	0.33(0.28, 0.37)	0.46(0.38, 0.55)	1.88(1.69, 2.09)
	Mexican American	1.03(0.90, 1.18)	1.52(1.30, 1.78)	0.87(0.77, 0.97)	1.14(1.01, 1.29)	0.58(0.47, 0.71)	0.79(0.69, 0.90)
	Non-Hispanic Asian	0.92(0.75, 1.13)	1.32(1.07, 1.62)	1.03(0.87, 1.21)	0.97(0.77, 1.21)	0.45(0.35, 0.57)	0.90(0.73, 1.11)
	Other race	1.36(1.11, 1.66)	1.80(1.48, 2.18)	1.15(0.95, 1.39)	1.29(1.07, 1.55)	0.51(0.41, 0.65)	1.15(0.97, 1.37)
Marital status							
	No	1(reference)	1(reference)	1(reference)	1(reference)	1(reference)	1(reference)
	Yes	1.09(0.96, 1.22)	1.03(0.93, 1.13)	1.17(1.07, 1.28)	1.06(0.95, 1.18)	1.13(0.96, 1.31)	0.97(0.88, 1.06)
Education level							
	Less than high school	1(reference)	1(reference)	1(reference)	1(reference)	1(reference)	1(reference)
	High school graduate	1.00(0.86, 1.16)	0.96(0.83, 1.11)	0.96(0.83, 1.11)	1.00(0.89, 1.14)	0.99(0.83, 1.19)	0.99(0.86, 1.15)
	Some college	0.88(0.76, 1.02)	0.84(0.73, 0.97)	0.91(0.79, 1.04)	1.02(0.89, 1.17)	0.98(0.81, 1.20)	0.83(0.72, 0.94)
	College graduate or above	0.70(0.59, 0.82)	0.79(0.68, 0.93)	0.79(0.68, 0.92)	0.86(0.73, 1.02)	0.94(0.75, 1.17)	0.61(0.52, 0.71)
Insurance status							
	Uninsured	1(reference)	1(reference)	1(reference)	1(reference)	1(reference)	1(reference)
	Any Insurance	1.28(1.13, 1.45)	1.16(1.02, 1.32)	1.19(1.06, 1.33)	1.00(0.89, 1.12)	1.37(1.14, 1.64)	1.08(0.96, 1.22)
Income-to-poverty ratio							
	< 1	1(reference)	1(reference)	1(reference)	1(reference)	1(reference)	1(reference)
	1–2	0.98(0.85, 1.12)	0.95(0.83, 1.08)	0.85(0.74, 0.98)	1.09(0.95, 1.25)	1.04(0.85, 1.27)	1.05(0.92, 1.19)
	2–3.5	0.86(0.74, 1.01)	0.91(0.79, 1.05)	0.85(0.74, 0.98)	0.94(0.81, 1.10)	0.85(0.69, 1.04)	1.00(0.86, 1.17)
	≥3.5	0.82(0.69, 0.98)	0.85(0.73, 0.99)	0.78(0.67, 0.91)	0.97(0.83, 1.14)	0.74(0.61, 0.89)	1.03(0.87, 1.21)
Alcohol							
	No	1(reference)	1(reference)	1(reference)	1(reference)	1(reference)	1(reference)
	Yes	0.77(0.69, 0.86)	0.97(0.86, 1.09)	0.74(0.65, 0.84)	0.89(0.80, 0.99)	0.92(0.78, 1.10)	0.80(0.72, 0.89)
Smoking status							
	Never smoked	1(reference)	1(reference)	1(reference)	1(reference)	1(reference)	1(reference)
	Former smoker	1.20(1.07, 1.36)	1.11(0.97, 1.26)	1.03(0.94, 1.13)	1.19(1.06, 1.34)	1.27(1.08, 1.49)	1.03(0.93, 1.15)
	Current smoker	1.27(1.11, 1.45)	1.04(0.92, 1.17)	1.55(1.38, 1.75)	1.49(1.31, 1.70)	1.11(0.92, 1.35)	0.84(0.74, 0.95)
Activity							
	No	1(reference)	1(reference)	1(reference)	1(reference)	1(reference)	1(reference)
	Moderate	0.90(0.80, 1.02)	0.90(0.81, 1.01)	0.91(0.82, 1.02)	0.92(0.83, 1.02)	0.87(0.74, 1.02)	1.05(0.93, 1.17)
	Vigorous	0.53(0.47, 0.61)	0.62(0.55, 0.71)	0.70(0.62, 0.79)	0.67(0.59, 0.76)	0.54(0.47, 0.62)	0.80(0.70, 0.91)
BMI (kg/m^2^) ^a^							
	Normal weight (18.5–24.9)	1(reference)	1(reference)	1(reference)	1(reference)	1(reference)	1(reference)
	Overweight (25.0–29.9)	4.44(3.88, 5.09)	2.01(1.77, 2.28)	2.07(1.87, 2.31)	2.60(2.31, 2.93)	27.55(22.69, 33.45)	1.33(1.19, 1.48)
	Obesity (≥30.0)	16.49(14.20, 19.14)	4.79(4.22, 5.44)	3.76(3.33, 4.24)	4.10(3.63, 4.63)	1136.18(867.74, 1487.66)	2.14(1.91, 2.39)

^a ^BMI < 18.5 kg/m^2^ were excluded; BMI, body mass index

### Change in the distribution of characteristics among MetS

From 1999 to 2018, the distribution of age and sex, race, marital status, PIR, and smoking status among NHANES participants with MetS remained stable (all *P* > 0.05), whereas the education, insurance status, alcohol, and activity were changed (all *P* <  0.05) (Additional file 10 sTable 9; Additional file 1 sFigure 1). Those with a college degree raised from 13.19 to 22.90% from 1999 to 2018. The proportion of participants who had any insurance increased from 85.23 to 89.63% from 1999 to 2001 to 2017–2018. The percentage of alcohol raised from 65.30 to 92.84% during the study period. Details on the changes in the characteristics of MetS components were provided in Additional file 11 sTable 10 to Additional file 15 sTable 14.

### Trend in proportion of intervention and control among MetS

Among MetS, the total proportion of glucose-lowering, blood lipid regulation, blood pressure lowering, and vigorous activity were 20.83, 37.73, 12.34, and 15.41%, respectively (Table 3). From 1999 to 2018, the use of glucose-lowering and blood-lipid regulation medication increased from 9.87 to 28.63% and from 16.16 to 45.11%, respectively; while vigorous activity decreased from 23.79 to 16.53% (all *P* for trend < 0.05) (Table 2; Fig. 2 B). The use of blood pressure-lowering medication raised from 9.58 to 15.57% from 1999 to 2000 to 2009–2010 and gradually fell to 9.65% after then. The trends in the intervention of glucose-lowering, blood-lipid regulation, blood pressure-lowering, and physical activity in the subgroup population were shown in Additional file 16 sTable 15- Additional file 19 sTable 18.

**Table 3:** Trends in the proportion of intervention and control among metabolic syndrome from 1999 to 2018

Characteristics			Total (*n* = 7733)	1999–2000 (*n* = 663)	2001–2002 (*n* = 739)	2003–2004 (*n* = 659)	2005–2006 (*n* = 615)	2007–2008 (*n* = 850)	2009–2010 (*n* = 896)	2011–2012 (*n* = 754)	2013–2014 (*n* = 824)	2015–2016 (*n* = 833)	2017–2018 (*n* = 900)	AAPC	*P* for trend
Intervention	Glucose lowering		20.83(19.08,22.59)	9.87(5.64, 14.11)	11.98(8.94, 15.01)	14.67(10.90, 18.45)	18.24(14.52, 21.96)	23.22(19.14, 27.30)	20.76(17.77, 23.76)	23.97(17.79, 30.16)	22.66(19.04, 26.29)	26.84(22.34, 31.34)	28.63(23.86, 33.40)	4.671(2.896, 6.477)	< 0.001
	Blood lipid regulation		37.73(35.23,40.22)	16.16(10.92, 21.40)	24.14(21.07, 27.22)	32.82(28.28, 37.37)	38.22(33.35, 43.08)	40.27(36.46, 44.09)	39.85(35.42, 44.29)	42.43(36.86, 48.01)	44.55(40.70, 48.40)	43.45(39.79, 47.12)	45.11(38.98, 51.25)	3.290(1.433, 5.182)	0.003
	Blood pressure lowering		12.34(10.99,13.70)	9.58(4.07, 15.08)	10.55(7.29, 13.80)	13.97(9.92, 18.02)	13.13(8.16, 18.09)	15.27(11.41, 19.12)	15.57(11.98, 19.16)	12.45(8.57, 16.33)	12.94(9.51, 16.38)	10.73(7.13, 14.34)	9.65(6.83, 12.47)	−0.797(−3.354, 1.827)	0.500
	Physical activity		47.41(44.63, 50.19)	51.00(43.19, 58.81)	55.31(50.08, 60.54)	62.22(57.01, 67.43)	58.25(53.55, 62.96)	39.51(32.87, 46.16)	40.27(37.47, 43.08)	43.64(36.59, 50.69)	39.05(35.15, 42.95)	42.54(37.34, 47.74)	47.48(41.18, 53.77)	−2.451(−4.452,-0.408)	0.025
	Moderate activity		31.99(29.65,34.34)	27.21(19.13, 35.29)	32.95(27.28, 38.62)	44.81(40.83, 48.79)	32.80(27.19, 38.40)	31.03(26.23, 35.83)	30.37(26.92, 33.82)	32.24(24.42, 40.06)	28.45(24.98, 31.91)	30.31(26.71, 33.91)	30.94(24.41, 37.47)	−2.116(−4.151, −0.038)	0.047
	Vigorous activity		15.41(14.11,16.72)	23.79(19.41, 28.17)	22.36(17.54, 27.19)	17.41(13.48, 21.34)	25.46(20.71, 30.21)	8.48(5.25, 11.71)	9.90(6.52, 13.29)	11.40(6.97, 15.83)	10.61(7.97, 13.24)	12.23(9.67, 14.79)	16.53(13.02, 20.05)	−3.565(−6.756, −0.264)	0.038
Control	HbA1c	< 8 %	92.95(88.30,97.59)	91.32(88.32, 94.32)	95.62(94.06, 97.18)	96.34(94.41, 98.27)	93.55(91.16, 95.94)	93.69(92.11, 95.26)	95.18(93.14, 97.22)	89.63(85.27, 93.99)	90.30(88.32, 92.28)	92.48(90.11, 94.85)	93.34(91.37, 95.31)	−0.113(−0.808, 0.587)	0.75
		< 7 %	86.20(81.85,90.56)	87.13(83.00, 91.26)	91.17(88.69, 93.66)	90.80(86.87, 94.73)	88.17(85.29, 91.05)	87.23(84.69, 89.77)	85.76(82.22, 89.31)	83.91(78.52, 89.29)	84.53(81.96, 87.11)	83.23(79.32, 87.14)	84.06(79.91, 88.21)	−0.509(− 0.724, − 0.293)	0.001
	Fasting blood-glucose	< 5.6 mmol/L	38.00(35.43,40.57)	46.22(37.83, 54.61)	49.56(45.87, 53.25)	46.24(41.08, 51.41)	38.09(33.68, 42.49)	37.94(31.48, 44.39)	38.07(34.46, 41.68)	34.12(30.08, 38.15)	34.73(29.81, 39.65)	32.50(26.40, 38.60)	30.40(24.03, 36.77)	−2.922(−4.218, −1.609)	< 0.001
	HDL	≥1.3 mmol/L	44.48(41.81,47.15)	27.28(23.05, 31.52)	35.13(31.29, 38.97)	43.84(39.45, 48.22)	52.92(48.26, 57.58)	45.38(42.34, 48.41)	41.84(37.90, 45.78)	50.32(42.90, 57.75)	44.98(39.98, 49.98)	47.65(42.48, 52.82)	48.89(43.59, 54.20)	3.113(−0.874, 7.26)	0.128
	Triglyceride	< 1.7 mmol/L	39.80(37.22,42.37)	32.97(26.34, 39.60)	31.77(28.97, 34.57)	38.98(31.54, 46.42)	37.92(32.20, 43.64)	38.07(32.94, 43.21)	45.68(41.06, 50.30)	39.30(29.76, 48.85)	42.36(37.15, 47.57)	40.74(35.85, 45.64)	45.54(39.55, 51.54)	1.935(0.832, 3.05)	0.004
	BMI ^a^	< 25 kg/m^2^	7.87(7.01, 8.74)	11.36(6.21, 16.52)	10.38(7.98, 12.77)	9.97(7.28, 12.67)	6.39(4.24, 8.53)	6.63(4.63, 8.62)	7.33(4.69, 9.97)	8.56(6.00, 11.12)	6.65(3.81, 9.50)	5.63(4.14, 7.12)	7.49(5.77, 9.21)	−2.485(−4.531, −0.395)	0.026
	Blood pressure	< 140/90 mmHg	69.28(65.58,72.98)	62.96(58.07, 67.85)	65.47(61.69, 69.24)	68.08(64.58, 71.58)	66.07(61.29, 70.85)	71.43(67.53, 75.32)	74.84(71.48, 78.20)	71.92(68.88, 74.97)	73.20(66.91, 79.49)	69.15(64.93, 73.38)	69.13(65.73, 72.53)	0.481(−0.028, 0.993)	0.064
		< 130/80 mmHg	40.03(37.22,42.84)	33.67(27.95, 39.39)	32.12(27.44, 36.81)	36.03(29.57, 42.49)	34.94(29.12, 40.75)	45.01(38.65, 51.36)	47.17(41.22, 53.11)	43.08(37.54, 48.62)	44.92(39.93, 49.90)	41.89(36.84, 46.94)	39.68(33.21, 46.15)	1.584(0.154, 3.036)	0.034

^a^ BMI < 18.5 kg/m^2^ were excluded; BMI, body mass index

During the whole survey intervals, the control percentages of Hb1Ac (< 7%), fasting blood glucose (< 5.6 mmol/L), HDL (≥1.3 mmol/L), triglyceride (< 1.7 mmol/L), BMI (<25 kg/m^2^) and blood pressure (< 130 mmHg) were 86.20, 38.00, 44.48, 39.80, 7.87, and 40.03%, respectively (Table 2). The percentages of participants who achieved Hb1Ac < 7% and fasting blood glucose < 5.6 mmol/L declined from 87.13 to 84.06% and from 46.22 to 30.40% (all *P* for trend < 0.05) through the study period (Table 2; Fig. 2 C). Opposite trends were observed for triglyceride control (< 1.7 mmol/L) and blood pressure control (< 130 mmHg) from 32.97 to 45.54% and from 33.67 to 39.68% (all *P* for trend < 0.05). The normal BMI (<25 kg/m^2^) displayed a downward trend from 11.36 to 7.49% (all *P* for trend < 0.05). The trends in the control of HbA1c, triglyceride, BMI, and blood pressure in the subgroup population were shown in Additional file 20 sTable 19-Additional file 23 sTable 22.

### Analysis of influence factors for control of MetS after intervention

Subsequently, we explored the influence factors of metabolic risk-factors control in MetS after intervention (Table 4). 45–64 years old, and male increased the risk of Hb1Ac uncontrol (≥7%) in persons with glucose-lowering medication among MetS. The younger (aged 20–44 years old), non-Hispanic white, and smoking now were the risk factors for uncontrol of HDL(< 1.3 mmol/L) and triglyceride (≥1.7 mmol/L) in persons with lipid-regulating medication. The uncontrol of BMI (> 25 kg/m^2^) among persons with vigorous or moderate activity was raised mainly in the population of younger (aged 20–44 years old), male, non-Hispanic black, middle income (PIR 1–3.5) and smoking (former and current). The risk of blood pressure uncontrol in persons with blood pressure-lowering medication was similar in most subpopulations, except for the reduced risk of high income (PIR ≥ 3.5).

**Table 4:** Analysis of influence factors for controlled of MetS after intervention

Factors		Hb1Ac ≥ 7% in persons with glucose-lowering medication	HDL < 1.3 mmol/L in persons with lipid-regulating medication	TG ≥ 1.7 mmol/L in persons with lipid-regulating medication	BMI > 25 kg/m^2^ in persons with vigorous or moderate activity ^a^	Blood Pressure of ≥140/90 mmHg in persons with blood pressure-lowering medication
Age (years)						
	20–44	1(reference)	1(reference)	1(reference)	1(reference)	1(reference)
	45–64	0.60(0.36,0.98)	1.37(0.80,2.34)	1.28(0.78,2.11)	2.23(1.15, 4.32)	1.12(0.52,2.42)
	≥65	1.07(0.62,1.83)	1.86(1.07,3.23)	2.09(1.23,3.55)	5.55(2.91,10.60)	0.93(0.45,1.94)
Sex						
	Female	1(reference)	1(reference)	1(reference)	1(reference)	1(reference)
	Male	0.60(0.46,0.78)	1.44(1.04,1.98)	1.23(0.97,1.55)	0.40(0.25, 0.64)	1.06(0.71,1.59)
Race and ethnicity						
	Non-Hispanic white	1(reference)	1(reference)	1(reference)	1(reference)	1(reference)
	Non-Hispanic black	0.91(0.65,1.27)	1.81(1.31,2.48)	4.09(2.97,5.63)	0.45(0.25, 0.79)	0.79(0.54,1.15)
	Mexican American	0.68(0.44,1.06)	1.42(1.04,1.94)	0.87(0.64,1.17)	0.57(0.27, 1.21)	0.81(0.42,1.59)
	Non-Hispanic Asian	0.67(0.44,1.03)	0.71(0.44,1.13)	1.35(0.90,2.01)	1.04(0.41, 2.64)	0.59(0.26,1.35)
	Other race	0.72(0.39,1.33)	1.38(0.78,2.42)	1.24(0.73,2.11)	3.43(1.99, 5.92)	0.97(0.38,2.49)
Marital status						
	No	1(reference)	1(reference)	1(reference)	1(reference)	1(reference)
	Yes	1.13(0.83,1.54)	1.07(0.82,1.40)	0.98(0.75,1.27)	0.95(0.62, 1.45)	1.00(0.69,1.44)
Education level						
	Less than high school	1(reference)	1(reference)	1(reference)	1(reference)	1(reference)
	High school graduate	0.84(0.59,1.20)	1.22(0.88,1.67)	0.80(0.58,1.09)	0.95(0.54, 1.66)	0.74(0.46,1.20)
	Some college	1.15(0.78,1.71)	1.46(1.04,2.05)	0.83(0.58,1.19)	0.71(0.39, 1.27)	0.97(0.59,1.58)
	College graduate or above	0.89(0.56,1.40)	1.03(0.69,1.55)	0.93(0.64,1.35)	0.91(0.47, 1.76)	1.06(0.62,1.81)
Insurance status		1.36(0.82,2.26)	1.13(0.70,1.84)	1.50(0.90,2.49)	0.95(0.48, 1.88)	0.54(0.27,1.07)
	Uninsured	1(reference)	1(reference)	1(reference)	1(reference)	1(reference)
	Any Insurance	1.36(0.82,2.26)	1.13(0.70,1.84)	1.50(0.90,2.49)	0.95(0.48, 1.88)	0.54(0.27,1.07)
Income-to-poverty ratio						
	< 1	1(reference)	1(reference)	1(reference)	1(reference)	1(reference)
	1–2	1.07(0.68,1.67)	0.89(0.63,1.24)	0.76(0.51,1.13)	0.47(0.26, 0.85)	1.06(0.64,1.76)
	2–3.5	0.90(0.55,1.47)	0.93(0.63,1.39)	0.83(0.55,1.24)	0.49(0.27, 0.91)	1.38(0.78,2.47)
	≥3.5	1.37(0.85,2.22)	1.05(0.70,1.60)	0.79(0.53,1.17)	0.55(0.30, 1.01)	1.94(1.07,3.52)
Alcohol						
	No	1(reference)	1(reference)	1(reference)	1(reference)	1(reference)
	Yes	1.22(0.88,1.68)	1.10(0.86,1.41)	1.17(0.90,1.52)	1.23(0.80, 1.90)	0.94(0.63,1.38)
Smoking status						
	Never smoked	1(reference)	1(reference)	1(reference)	1(reference)	1(reference)
	Former smoker	0.87(0.63,1.20)	0.82(0.63,1.07)	0.76(0.59,0.99)	0.87(0.57, 1.33)	1.27(0.86,1.88)
	Current smoker	1.02(0.64,1.62)	0.53(0.36,0.78)	0.67(0.46,0.98)	1.81(0.99, 3.30)	1.59(0.96,2.61)
Activity						
	No	1(reference)	1(reference)	1(reference)	–	1(reference)
	Moderate	0.92(0.66,1.30)	1.08(0.83,1.41)	1.15(0.90,1.46)	–	0.83(0.53,1.30)
	Vigorous	1.22(0.67,2.20)	1.61(1.01,2.54)	1.31(0.85,2.03)	–	0.88(0.44,1.76)
BMI (kg/m^2^) ^a^						
	Normal weight (18.5–24.9)	1(reference)	1(reference)	1(reference)	–	1(reference)
	Overweight (25.0–29.9)	1.19(0.70,2.03)	0.84(0.57,1.23)	0.96(0.64,1.44)	–	1.10(0.53,2.29)
	Obesity (≥30.0)	0.99(0.58,1.71)	0.64(0.45,0.91)	0.86(0.60,1.23)	–	1.14(0.58,2.25)

^a^ BMI < 18.5 were excluded; BMI, body mass index

## Discussion

**Fig. 3:**
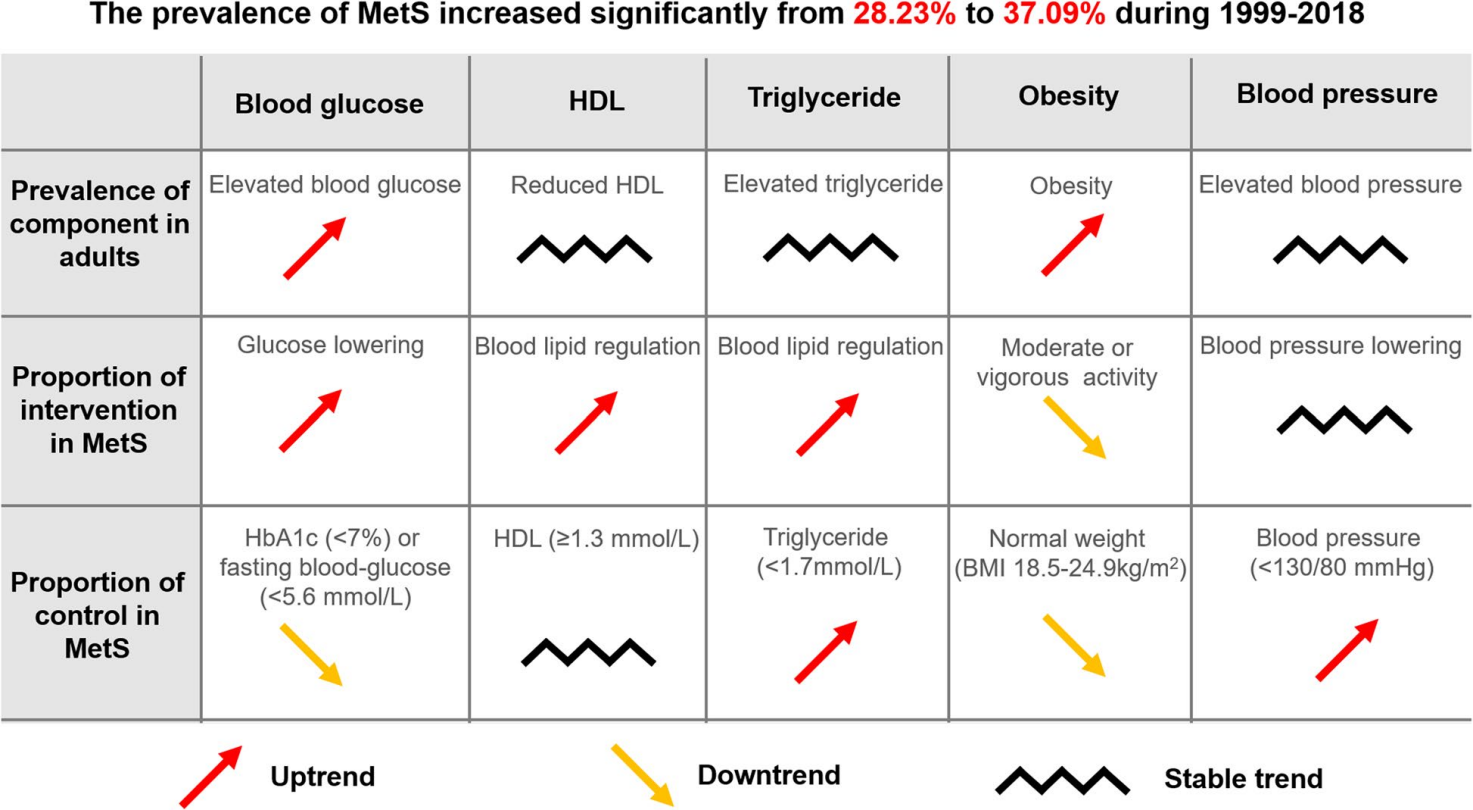
Summary of the trend in prevalence of intervention and control among metabolic syndrome from 1999 to 2018

The estimated prevalence of MetS and its components of elevated blood glucose and obesity among US adults increased significantly between 1999 and 2000 and 2017–2018, paralleling a recent rise in the prevalence of diabetes [8] and obesity [11]. There were increase in the use of blood glucose-lowering and decrease in the intervention of vigorous activity, accompanied by a descent in the control of blood-glucose and obesity among MetS during the whole period (Fig. 3). Therefore, elevated blood glucose and obesity are the main causes of MetS burden, which is the focus of prevention and control. Quitting smoking and increasing physical activity may decrease the prevalence of MetS. Ultimately, we screened out the focus population of uncontrol for blood-glucose and obesity, whom should be modified treatment and improved lifestyle in the management of MetS.

The significant increasing trend in the estimated prevalence of MetS mainly consists of four reasons. Firstly, with the convenience and availability of testing in blood glucose, blood lipid, blood pressure, and waist circumference, an increasing number of apparently healthy people could be diagnosed with MetS, leading to the augment of prevalence. Secondly, the increased trend in MetS prevalence may in part be a collective product of improved survival in diabetes, dyslipidemia, hypertension, and obesity, due to the advance in clinical treatment [27–30] and management policy [31, 32]. The *Global action plan for the prevention and control of non-communicable diseases 2013–2020* is proposed by WHO aimed to reduce the preventable and avoidable burden of morbidity, mortality, and disability due to noncommunicable diseases (including cardiovascular diseases and diabetes) [31]. Correspondingly, America put forward to *Plan of Action for the Prevention and Control of Noncommunicable Diseases for 2012–2025* [32], contributing to the chronic duration and longer life span in MetS. Thirdly, the burden of MetS among children and adolescents may result in the increasing prevalence of MetS in adults. A systematic review with modeling analysis revealed that the global prevalence of MetS in 2020 at 2.8% for children and 4.8% for adolescents [33], who may become the adult MetS with age growth. Last and most important, new cases were the direct cause of the increased prevalence of MetS. Our study found that the major subpopulations with increased MetS prevalence were the older, male, non-Hispanic white, low level of education and income, any insurance, BMI ≥ 25 kg/m^2^, smoking and no activity, which may construct the source of new cases.

The above factors may be the risk factors for the prevalence of MetS. With an aging US population and accumulation of cases, prevalence among those aged 65 years or older remained high and presented an increasing trend, consistent with previous studies [15]. There was an identical risk of MetS in non-Hispanic white and Mexican American but the reduced risk in non-Hispanic black and female, accompanied by the increasing prevalence trend of MetS in non-Hispanic white and Mexican American, which may be related to the differences of race and gender in metabolic status [34]. Our result manifested that highly educated (college graduate or above) and high-income (PIR ≥ 3.5) had lower risks of MetS. Meanwhile, a higher prevalence of MetS and an increasing trend were observed in the insurance subpopulation. The individuals with insurance may reflect a higher state of economic condition, who were more accessible to physical examination and aware of the MetS; while persons with high education and income probably know more about health knowledge to avoid metabolic risk factors [35]. Our data showed a lower risk of MetS in alcoholic adults and an increasing prevalence trend of MetS in alcoholic adults. The reversal appearance may be related to the increase in the proportion of people who drink alcohol among MetS. Drinking alcohol is a double-edged sword for MetS due to the amount and type of alcohol consumed [36]. In this study, alcohol meant having at least 12 drinks of any alcoholic beverage in the past 12 months or lifetime, which may contain a quantity of moderate drinking, associating with a reduced risk of MetS. Nevertheless, the proportion of alcohol among MetS raised, which may be respect with an annual increment of 0.2% for alcohol-drinking prevalence [37]. The risk of MetS was significantly higher in either former or current smokers, conforming with the previous investigation [38]. We found that the risk of MetS decreased with vigorous activity (not moderate activity), in conformity with previous research [39], in which underlying mechanisms were the improvement of insulin resistance, acceleration of fat metabolism, and alleviation of inflammation response [40]. Accordingly, quitting smoking and increasing physical activity may decrease the prevalence of MetS.

Since the trend in the prevalence of MetS is increasing, it is necessary to understand the intervention and control of MetS to provide direction for prevention and control in the future. Our data showed that the use of glucose-lowering medication was increasing, while the control of blood glucose declined among MeS, accompanied by the raised prevalence trend of elevated blood glucose in adults from 1999 to 2018. This inverse change between hypoglycemic drug use and blood glucose control was corresponding to the researches on diabetes [7, 8]. We discovered that the 45–64 years old, and male were the focus population of Hb1Ac uncontrol (≥7%) in persons with glucose-lowering medication among MetS. On the one hand, it may indicate that the treatment strategy is inappropriate in this population and needs to be adjusted; on the other hand, drug therapy alone cannot control blood glucose well in older and male who should improve lifestyle intensively, such as physical activity. Studies have proved that activity enhancement and weight loss have beneficial effects on the control of blood glucose, blood lipids, and blood pressure [41–43]. However, we found decreasing in moderate or vigorous activity among MeS, with an uptrend trend in the prevalence of obesity. It is stated that current intervention of antidiabetic and physical activity is insufficient in the control of blood glucose and obesity. Therefore, the focus population should be screened out and strengthened management in the control of blood glucose and obesity. According to our findings, the focus population were the older and male for blood glucose control; the younger, male, non-Hispanic black, middle income, and smoking for BMI control.

Our study had several strengths. First, we updated the prevalence of MetS nationally represented US adults, and estimated the tendency of MetS in the subgroup of social demography and lifestyle. Second, this work extends prior findings by considering the trend in prevalence of MetS, and the tendency in proportion of intervention and control among MetS. The increasing trend of MetS is mainly reflected in the enhancement of elevated blood glucose and obesity, with the consistency tendency of glucose and weight control among US adults during 1999–2018. Third, based on the epidemiological characteristics of MetS, we explored the influencing factors of its prevalence and control to provide theoretical basis for the health policy.

This study has several limitations. Firstly, there are non-response bias, measurement bias, and recall bias during the whole survey period in NHANES. The unweighted response rates ranged from 52 to 84% for the household interview and 49 to 80% for the medical examination during 1999–2018 [7]. The change of blood biochemistry analyzer and the variation of HDL measuring method may be referred to the measurement bias. Besides, data on medication use may be subject to recall bias. Secondly, the type and number of anti-diabetics, anti-hypertensive and regulate-lipids drugs were not subdivided; thus, the relationship between concrete drug use strategy, MetS control, and epidemic trend cannot be entirely certain. Meanwhile, most lipid-regulating drugs can decrease triglyceride and increase HDL synchronously; therefore, we cannot distinguish drugs for elevated triglyceride and reduced HDL accurately. Thirdly, for the analysis of influencing factors, the direction of causal correlation may be uncertain, owing to the unknown of previous MetS conditions in this cross-sectional study. Fourth, we did not include dietary related variables, which may overlook the trend in the prevalence of dietary subgroups and the effects of diet on MetS. Fifth, the reasons for the contradiction phenomenon that the trend of antidiabetic use increased and the tendency of glycemic control decreased have not been fully illuminated, which should be further studied.

## Conclusion

The estimated and prevalence of MetS increased significantly between 1999 and 2000 and 2017–2018, especially in elevated blood glucose and obesity. Quitting smoking and increasing degree of physical activity may be the effective measurement to reduce the prevalence of MetS. There was uptrend of antidiabetic and downtrend of the moderate or vigorous activity, accompanied by declined controls of blood glucose and obesity. In the control of blood-glucose and obesity, we should screen out the focus population to modify treatment and improve lifestyle.

## Supplementary Information

**Additional file 1.** sFigure 1 Change in distribution of characteristics among metabolic syndrome from 1999 to 2002 to 2015–2018.

**Additional file 2.** sTable 1 The sample size of each 2-year survey circle in this Study.

**Additional file 3.** sTable 2 Annual prevalence change of metabolic syndrome and components among US adults, 1999–20,118.

**Additional file 4.** sTable 3 Trend in prevalence of elevated blood glucose among US adults from 1999 to 2018.

**Additional file 5.** sTable 4 Trend in prevalence of reduced HDL among US adults from 1999 to 2018.

**Additional file 6.** sTable 5 Trend in prevalence of elevated triglyceride among US adults from 1999 to 2018.

**Additional file 7.** sTable 6 Trend in prevalence of obesity among US adults from 1999 to 2018.

**Additional file 8.** sTable 7 Trend in prevalence of elevated blood pressure among US adults from 1999 to 2018.

**Additional file 9.** sTable 8 Prevalence of metabolic syndrome and components among US adults from 1999 to 2018.

**Additional file 10.** sTable 9 Change in the distribution of characteristics among metabolic syndrome from 1999 to 2018.

**Additional file 11.** sTable 10 Change in distribution of characteristics among elevated blood glucose from 1999 to 2018.

**Additional file 12.** sTable 11 Change in distribution of characteristics among reduced HDL from 1999 to 2018.

**Additional file 13.** sTable 12 Change in distribution of characteristics among elevated triglyceride from 1999 to 2018.

**Additional file 14.** sTable 13 Change in distribution of characteristics among obesity from 1999 to 2018.

**Additional file 15.** sTable 14 Change in distribution of characteristics among elevated blood pressure from 1999 to 2018.

**Additional file 16.** sTable 15 Trend in treatment of glucose lowering medication among metabolic syndrome from 1999 to 2018.

**Additional file 17.** sTable 16 Trend in treatment of blood-lipid regulated medication among metabolic syndrome from 1999 to 2018.

**Additional file 18.** sTable 17 Trend in intervention of physical activity among metabolic syndrome from 1999 to 2018.

**Additional file 19.** sTable 18 Trend in treatment of blood pressure lowering medication among metabolic syndrome from 1999 to 2018.

**Additional file 20.** sTable 19 Trend in control rate of HbA1camong metabolic syndrome from 1999 to 2018.

**Additional file 21.** sTable 20 Trend in control rate of triglyceride among metabolic syndrome from 1999 to 2018.

**Additional file 22.** sTable 21 Trend in control rate of weight status among metabolic syndrome from 1999 to 2018.

**Additional file 23.** sTable 22 Trend in control rate of blood pressure among metabolic syndrome from 1999 to 2018.

## Data Availability

The data were publicly available from NHANSE (https://www.cdc.gov/nchs/nhanes/). All data generated or analyzed during this study are included in this published article.

## Abbreviations

MetS: metabolic syndrome
NHANES: National Health and Nutrition Examination Survey
MEC: mobile examination center
PSUs: primary sampling units
ATP III: Adult Treatment Panel III
HDL: high-density lipoprotein
BMI: body mass index
PIR: income-to-poverty ratio
CI: confidence interval
OR: odds ratio.

## References

[1] Eckel RH, Alberti KG, Grundy SM, Zimmet PZ (2010). The metabolic syndrome. Lancet.

[2] Mongraw-Chaffin M, Foster MC, Anderson C, Burke GL, Haq N, Kalyani RR, Ouyang P, Sibley CT, Tracy R, Woodward M (2018). Metabolically Healthy Obesity, Transition to Metabolic Syndrome, and Cardiovascular Risk. J Am Coll Cardiol.

[3] DeBoer MD, Filipp SL, Gurka MJ (2018). Use of a Metabolic Syndrome Severity Z Score to Track Risk During Treatment of Prediabetes: An Analysis of the Diabetes Prevention Program. Dia Care.

[4] Zhang F, Liu L, Zhang C, Ji S, Mei Z, Li T (2021). Association of Metabolic Syndrome and Its Components With Risk of Stroke Recurrence and Mortality: A Meta-analysis. Neurology.

[5] Wu SH, Liu Z, Ho SC (2010). Metabolic syndrome and all-cause mortality: a meta-analysis of prospective cohort studies. Eur J Epidemiol.

[6] Tsao CW, Aday AW, Almarzooq ZI, Alonso A, Beaton AZ, Bittencourt MS, Boehme AK, Buxton AE, Carson AP, Commodore-Mensah Y (2022). Heart Disease and Stroke Statistics-2022 Update: A Report From the American Heart Association. Circulation.

[7] Fang M, Wang D, Coresh J, Selvin E (2021). Trends in Diabetes Treatment and Control in U.S. Adults, 1999–2018. N Engl J Med.

[8] Wang L, Li X, Wang Z, Bancks MP, Carnethon MR, Greenland P, Feng YQ, Wang H, Zhong VW (2021). Trends in Prevalence of Diabetes and Control of Risk Factors in Diabetes Among US Adults, 1999–2018. JAMA.

[9] Bucholz EM, Rodday AM, Kolor K, Khoury MJ, de Ferranti SD (2018). Prevalence and Predictors of Cholesterol Screening, Awareness, and Statin Treatment Among US Adults With Familial Hypercholesterolemia or Other Forms of Severe Dyslipidemia (1999–2014). Circulation.

[10] Zhou B, Carrillo-Larco RM, Danaei G, Riley LM, Paciorek CJ, Stevens GA, Gregg EW, Bennett JE, Solomon B, Singleton RK, Sophiea MK (2021). Worldwide trends in hypertension prevalence and progress in treatment and control from 1990 to 2019: a pooled analysis of 1201 population-representative studies with 104 million participants. Lancet.

[11] Hales CM, Carroll MD, Fryar CD, Ogden CL (2020). Prevalence of Obesity and Severe Obesity Among Adults: United States, 2017–2018. NCHS Data Brief.

[12] Ford ES, Giles WH, Dietz WH (2002). Prevalence of the metabolic syndrome among US adults: findings from the third National Health and Nutrition Examination Survey. JAMA.

[13] Palmer MK, Toth PP (2019). Trends in Lipids, Obesity, Metabolic Syndrome, and Diabetes Mellitus in the United States: An NHANES Analysis (2003–2004 to 2013–2014). Obesity.

[14] Beltran-Sanchez H, Harhay MO, Harhay MM, McElligott S (2013). Prevalence and trends of metabolic syndrome in the adult U.S. population, 1999–2010. J Am Coll Cardiol.

[15] Hirode G, Wong RJ (2020). Trends in the Prevalence of Metabolic Syndrome in the United States, 2011–2016. JAMA.

[16] O'Hearn M, Lauren BN, Wong JB, Kim DD, Mozaffarian D (2022). Trends and Disparities in Cardiometabolic Health Among U.S. Adults, 1999–2018. J Am Coll Cardiol.

[17] Chitrala KN, Hernandez DG, Nalls MA, Mode NA, Zonderman AB, Ezike N, Evans MK (2020). Race-specific alterations in DNA methylation among middle-aged African Americans and Whites with metabolic syndrome. Epigenetics-Us.

[18] Krijnen HK, Hoveling LA, Liefbroer AC, Bultmann U, Smidt N (2022). Socioeconomic differences in metabolic syndrome development among males and females, and the mediating role of health literacy and self-management skills. Prev Med.

[19] Mathew AV, Li L, Byun J, Guo Y, Michailidis G, Jaiswal M, Chen YE, Pop-Busui R, Pennathur S (2018). Therapeutic Lifestyle Changes Improve HDL Function by Inhibiting Myeloperoxidase-Mediated Oxidation in Patients With Metabolic Syndrome. Diabetes Care.

[20] NCHS. Centers for Disease Control and Prevention National Health and Nutrition Examination Survey. https://www.cdc.gov/nchs/nhanes/. Accessed April, 2022.

[21] NCHS. Centers for Disease Control and Prevention NCHS Research Ethics Review Board (ERB) Approval. https://www.cdc.gov/nchs/nhanes/irba98.htm. Accessed April, 2022.

[22] Grundy SM, Cleeman JI, Daniels SR, Donato KA, Eckel RH, Franklin BA, Gordon DJ, Krauss RM, Savage PJ, Smith SJ (2005). Diagnosis and management of the metabolic syndrome: an American Heart Association/National Heart, Lung, and Blood Institute Scientific Statement. Circulation.

[23] NCHS. National Health and Nutrition Examination Survey. Prescription medications, drug information. https://wwwn.cdc.gov/Nchs/Nhanes/1999-2000/RXQ_DRUG.htm. Accessed April, 2022.

[24] Clinical guidelines on the identification, evaluation, and treatment of overweight and obesity in adults: executive summary. Expert Panel on the Identification, Evaluation, and Treatment of Overweight in Adults. Am J Clin Nutr. 1998;68(4):899–917. 10.1093/ajcn/68.4.899 9771869

[25] Vieux F, Maillot M, Rehm CD, Drewnowski A (2020). Flavonoid Intakes in the US Diet Are Linked to Higher Socioeconomic Status and to Tea Consumption: Analyses of NHANES 2011–16 Data. J Nutr.

[26] Kim HJ, Fay MP, Feuer EJ, Midthune DN (2000). Permutation tests for joinpoint regression with applications to cancer rates. Stat Med.

[27] O'Malley PG, Arnold MJ, Kelley C, Spacek L, Buelt A, Natarajan S, Donahue MP, Vagichev E, Ballard-Hernandez J, Logan A (2020). Management of Dyslipidemia for Cardiovascular Disease Risk Reduction: Synopsis of the 2020 Updated U.S. Department of Veterans Affairs and U.S. Department of Defense Clinical Practice Guideline. Ann Intern Med.

[28] Improving Care and Promoting Health in Populations (2021). Standards of Medical Care in Diabetes-2021. Diabetes Care.

[29] Al-Makki A, DiPette D, Whelton PK, Murad MH, Mustafa RA, Acharya S, Beheiry HM, Champagne B, Connell K, Cooney MT (2022). Hypertension Pharmacological Treatment in Adults: A World Health Organization Guideline Executive Summary. Hypertension.

[30] Draznin B, Aroda VR, Bakris G, Benson G, Brown FM, Freeman R, Green J, Huang E, Isaacs D, Kahan S (2022). 8. Obesity and Weight Management for the Prevention and Treatment of Type 2 Diabetes: Standards of Medical Care in Diabetes-2022. Diabetes Care.

[31] WHO. Global Action Plan for the Prevention and Control of Non-Communicable Diseases 2013–2020. http://apps.who.int/iris/bitstream/handle/10665/94384/9789241506236_eng.pdf;jsessionid=82779261865AD81DFA61D751267388CA?sequence=1. Accessed April 9.

[32] PAHO W. Plan of action for the prevention and control of noncommunicable diseases. https://www.paho.org/hq/dmdocuments/2013/CD52-7-e.pdf. Accessed April 9.

[33] Noubiap JJ, Nansseu JR, Lontchi-Yimagou E, Nkeck JR, Nyaga UF, Ngouo AT, Tounouga DN, Tianyi FL, Foka AJ, Ndoadoumgue AL (2022). Global, regional, and country estimates of metabolic syndrome burden in children and adolescents in 2020: a systematic review and modelling analysis. Lancet Child Adolesc Health.

[34] Moore JX, Chaudhary N, Akinyemiju T (2017). Metabolic Syndrome Prevalence by Race/Ethnicity and Sex in the United States, National Health and Nutrition Examination Survey, 1988–2012. Prev Chronic Dis.

[35] He Y, Wu W, Wu S, Zheng HM, Li P, Sheng HF, Chen MX, Chen ZH, Ji GY, Zheng ZD (2018). Linking gut microbiota, metabolic syndrome and economic status based on a population-level analysis. Microbiome.

[36] Sun K, Ren M, Liu D, Wang C, Yang C, Yan L (2014). Alcohol consumption and risk of metabolic syndrome: a meta-analysis of prospective studies. Clin Nutr.

[37] Cheng HG, Kaakarli H, Breslau J, Anthony JC (2018). Assessing Changes in Alcohol Use and Alcohol Use Disorder Prevalence in the United States: Evidence From National Surveys From 2002 Through 2014. Jama Psychiat.

[38] Kim SW, Kim HJ, Min K, Lee H, Lee SH, Kim S, Kim JS, Oh B (2021). The relationship between smoking cigarettes and metabolic syndrome: A cross-sectional study with non-single residents of Seoul under 40 years old. Plos One.

[39] Sagawa N, Rockette-Wagner B, Azuma K, Ueshima H, Hisamatsu T, Takamiya T, El-Saed A, Miura K, Kriska A, Sekikawa A (2020). Physical activity levels in American and Japanese men from the ERA-JUMP Study and associations with metabolic syndrome. J Sport Health Sci.

[40] Myers J, Kokkinos P, Nyelin E. Physical Activity, Cardiorespiratory Fitness, and the Metabolic Syndrome. Nutrients. 2019;11(7). 10.3390/nu11071652 PMC6683051 31331009

[41] Hall ME, Cohen JB, Ard JD, Egan BM, Hall JE, Lavie CJ, Ma J, Ndumele CE, Schauer PR, Shimbo D (2021). Weight-Loss Strategies for Prevention and Treatment of Hypertension: A Scientific Statement From the American Heart Association. Hypertension.

[42] Zhang Y, Yang J, Ye J, Guo Q, Wang W, Sun Y, Zeng Q (2019). Separate and combined associations of physical activity and obesity with lipid-related indices in non-diabetic and diabetic patients. Lipids Health Dis.

[43] Carbone S, Del BM, Ozemek C, Lavie CJ (2019). Obesity, risk of diabetes and role of physical activity, exercise training and cardiorespiratory fitness. Prog Cardiovasc Dis.

