# Supplementary material for: Trends and influence factors in the prevalence, intervention, and control of metabolic syndrome among US adults, 1999–2018
Source: BMC Geriatr. 2022 Dec 19;22:979. doi: 10.1186/s12877-022-03672-6 (PMC9764589; doi:10.1186/s12877-022-03672-6)
Supplement: Supplementary file 1 — Additional file 1. sFigure 1 Change in distribution of characteristics among metabolic syndrome from 1999 to 2002 to 2015–2018. [file 12877_2022_3672_MOESM1_ESM.docx]

**Supplement Lists**

sFigure 1 Change in distribution of characteristics among metabolic syndrome from 1999-2002 to 2015-2018

sTable 1 The sample size of each 2-year survey circle in this Study

sTable 2 Annual prevalence change of metabolic syndrome and components among US adults, 1999-20118

sTable 3 Trend in prevalence of elevated blood glucose among US adults from 1999 to 2018

sTable 4 Trend in prevalence of reduced HDL among US adults from 1999 to 2018

sTable 5 Trend in prevalence of elevated triglyceride among US adults from 1999 to 2018

sTable 6 Trend in prevalence of obesity among US adults from 1999 to 2018

sTable 7 Trend in prevalence of elevated blood pressure among US adults from 1999 to 2018

sTable 8 Prevalence of metabolic syndrome and components among US adults from 1999 to 2018

sTable 9 Change in the distribution of characteristics among metabolic syndrome from 1999 to 2018

sTable 10 Change in distribution of characteristics among elevated blood glucose from 1999 to 2018

sTable 11 Change in distribution of characteristics among reduced HDL from 1999 to 2018

sTable 12 Change in distribution of characteristics among elevated triglyceride from 1999 to 2018

sTable 13 Change in distribution of characteristics among obesity from 1999 to 2018

sTable 14 Change in distribution of characteristics among elevated blood pressure from 1999 to 2018

sTable 15 Trend in treatment of glucose lowering medication among Metabolic Syndrome from 1999 to 2018

sTable 16 Trend in treatment of blood-lipid regulated medication among metabolic syndrome from 1999 to 2018

sTable 17 Trend in intervention of physical activity among metabolic syndrome from 1999 to 2018

sTable 18 Trend in treatment of blood pressure lowering medication among metabolic syndrome from 1999 to 2018

sTable 19 Trend in control rate of HbA1camong metabolic syndrome from 1999 to 2018

sTable 20 Trend in control rate of triglyceride among metabolic syndrome from 1999 to 2018

sTable 21 Trend in control rate of weight status among metabolic syndrome from 1999 to 2018

sTable 22 Trend in control rate of blood pressure among metabolic syndrome from 1999 to 2018

**sTable1-sTable22 were showed in excel.xlsx file.**


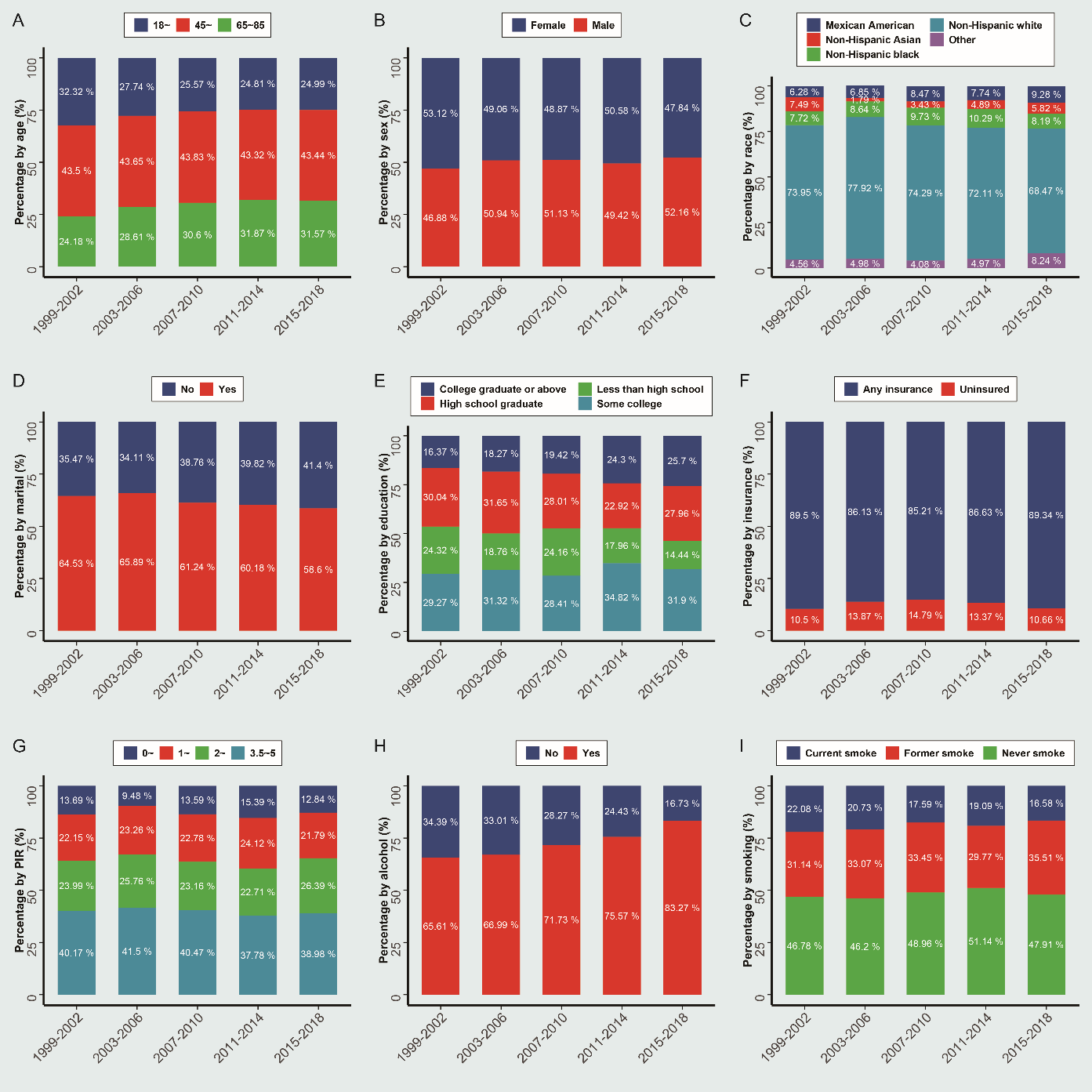


**sFigure 1 Change in distribution of characteristics among metabolic syndrome from 1999-2002 to 2015-2018.** Changes in distribution of age (B)**,** sex (C), race (D), marital status (E), education level (F), insurance status (G), PIR (H), alcohol (I) and smoking status (J) among metabolic syndrome. PIR, income-to-poverty ratio.
